# From coarse to fine: a deep 3D probability volume contours framework for tumour segmentation and dose painting in PET images

**DOI:** 10.3389/fradi.2023.1225215

**Published:** 2023-09-05

**Authors:** Wenhui Zhang, Surajit Ray

**Affiliations:** School of Mathematics and Statistics, University of Glasgow, Glasgow, United Kingdom

**Keywords:** image segmentation, PET imaging, probability volume contour, dose painting, deep learning

## Abstract

With the increasing integration of functional imaging techniques like Positron Emission Tomography (PET) into radiotherapy (RT) practices, a paradigm shift in cancer treatment methodologies is underway. A fundamental step in RT planning is the accurate segmentation of tumours based on clinical diagnosis. Furthermore, novel tumour control methods, such as intensity modulated radiation therapy (IMRT) dose painting, demand the precise delineation of multiple intensity value contours to ensure optimal tumour dose distribution. Recently, convolutional neural networks (CNNs) have made significant strides in 3D image segmentation tasks, most of which present the output map at a voxel-wise level. However, because of information loss in subsequent downsampling layers, they frequently fail to precisely identify precise object boundaries. Moreover, in the context of dose painting strategies, there is an imperative need for reliable and precise image segmentation techniques to delineate high recurrence-risk contours. To address these challenges, we introduce a 3D coarse-to-fine framework, integrating a CNN with a kernel smoothing-based probability volume contour approach (KsPC). This integrated approach generates contour-based segmentation volumes, mimicking expert-level precision and providing accurate probability contours crucial for optimizing dose painting/IMRT strategies. Our final model, named KsPC-Net, leverages a CNN backbone to automatically learn parameters in the kernel smoothing process, thereby obviating the need for user-supplied tuning parameters. The 3D KsPC-Net exploits the strength of KsPC to simultaneously identify object boundaries and generate corresponding probability volume contours, which can be trained within an end-to-end framework. The proposed model has demonstrated promising performance, surpassing state-of-the-art models when tested against the MICCAI 2021 challenge dataset (HECKTOR).

## 1. Introduction

Fluorodeoxyglucose Positron Emission Tomography (PET) has been widely recognized as an essential tool in oncology (1). Its applications in areas such as staging, monitoring, follow-up radiotherapy (RT) planning and therapy response assessment are rapidly growing in popularity (2–4). RT is an essential treatment method for malignant tumours. The process of delineating the gross tumour volume (GTV) in RT planning and radiomics analysis relies on manually annotating volumes of interest (VOIs) in three-dimensions, which allows extracting semi-quantitative metrics such as mean or maximum standardized uptake values (SUVs) (5).

PET has the potential to improve cancer therapy outcomes by enabling the identification and characterization of tumours based on their metabolic properties, which are closely linked to cancer biology (6). The quantitative assessment of the metabolically active tumour volume offers independent prognostic and predictive information, as evidenced by compelling data in various malignancies such as locally advanced esophageal cancer (7), lung cancer (8), cervical and head and neck cancers (9), non-Hodgkin lymphoma (10) and pleural mesothelioma (11). These promising results underscore the critical need to develop and validate robust algorithms for segmenting PET metabolic volumes before and during treatment.

The advent of multi-modality imaging technology has introduced combined PET-CT (computed tomography) and PET-MRI (magnetic resonance imaging), enabling the acquisition of both anatomical/morphological and functional information in a single imaging session. Nevertheless, the registration process of PET-CT or PET-MRI imaging modalities is often limited in its accuracy due to the differences in scanner, image acquisition, and reconstruction protocol (6). PET scans can be prone to various artifacts, such as respiratory motion, patient movement, and metal artifacts from implanted devices (12). These artifacts can affect the accuracy of the registration process and introduce uncertainties in aligning PET images with CT or MRI. Therefore, it is important to note that the primary objective of our research is to investigate the potential of utilizing metabolic information from PET scans to improve the accuracy of target delineation. By focusing on the metabolic characteristics provided by PET imaging, we aim to contribute to the development of novel methodologies that enhance the precision and interpretability of tumour segmentation in radiotherapy planning.

**Figure 1:**
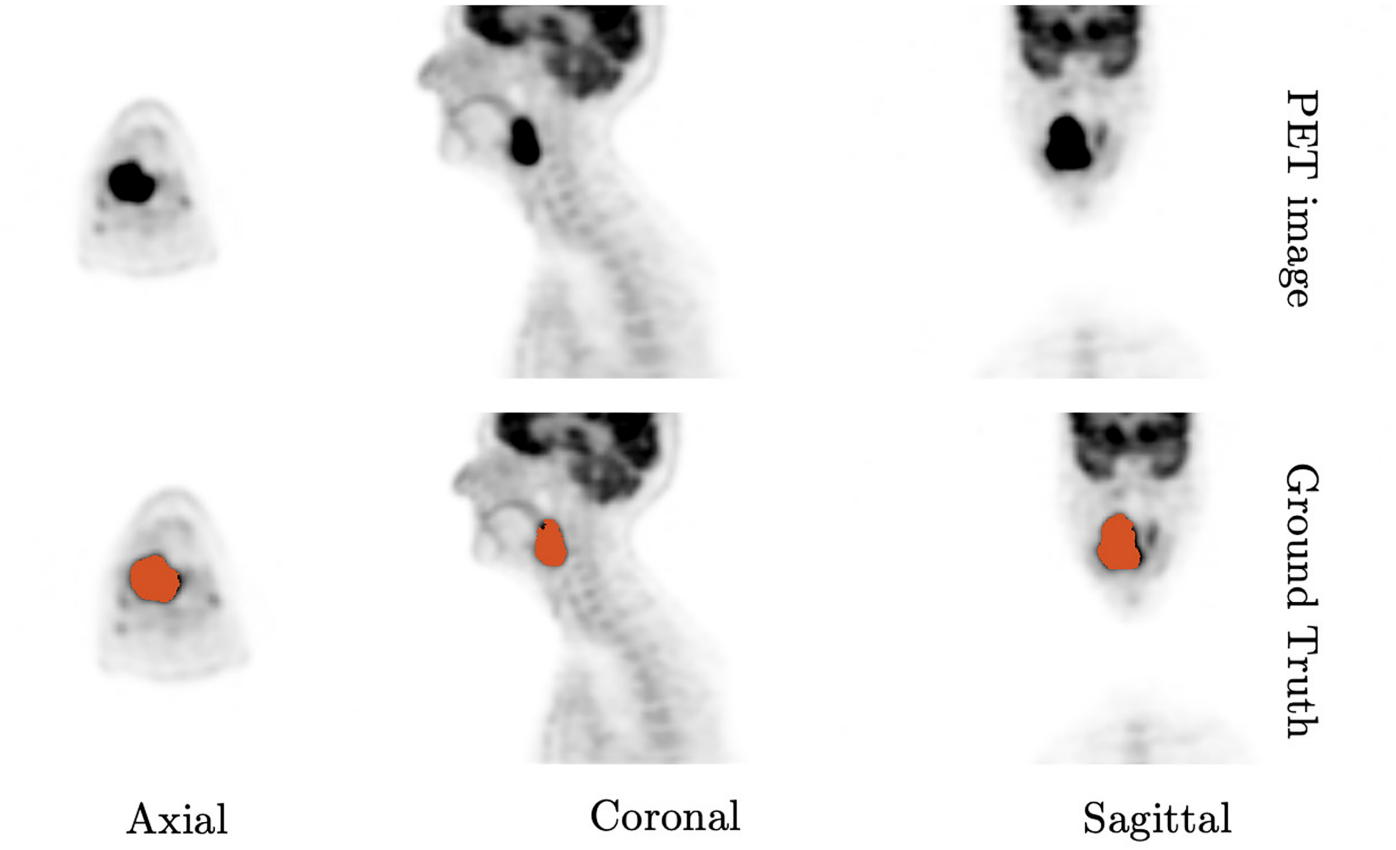
An case example of 3D PET scan from axial, coronal and sagittal angles on head and neck tumour. The orange annotations are provided by expert’s hand segmentation.

In the realm of 3D techniques, there exist various approaches for determining VOI, which can be categorized as either manual or automatic. Manual delineation for boundary definition is a time-consuming and subjective process (2), which can be prone to operator error and often leads to large inter-observer and intra-observer variations across different images and operators (13). An example of PET scan is shown in Figure 1. The task of automatic object segmentation in PET is more challenging, due to various factors such as low resolution, low contrast, and noise that can arise from radioactive decay or reconstruction methods (1). Therefore, developing highly accurate automatic segmentation algorithms for PET images is an urgent necessity to enable faster and more reproducible GTV definition, thus reducing the workload on experts and speeding up RT planning while reducing intra-observer variability. In addition, the utilization of fully automatic segmentation algorithms can greatly facilitate the practical application of validated models to patients’ images within standard clinical workflows. Beyond tumour delineation, another important use of functional images, such as PET images is their use for designing modulated radiation therapy (IMRT) dose painting. IMRT dose painting requires the accurate calculation of multiple nested contours of intensity values to optimise dose distribution across the tumour. Despite various segmentation strategies, there is a need to develop optimal image segmentation approaches that reproducibly and accurately identify the high recurrent-risk regions (14).

To address these issues, we propose a coarse-to-fine deep learning (DL) framework that can provide accurate segmentation results and produce probability volume contours designed to optimise dose painting/IMRT strategies as a byproduct. The rest of the paper is organized as follows. Section 2 covers the related work in PET image segmentation and corresponding dose painting strategies first. Then it highlights the motivations and novelties of the proposed model. The framework and network architecture of our proposed model with training details is described in Section 3. Section 4 presents the data information and the evaluation metrics used for model performance. At the beginning of Section 5, the experimental results of the proposed model are presented and discussed with comparisons to some state-of-art models. Following this, several visualization examples of the application of our proposed model in dose painting/IMRT are displayed and explained. The summary and conclusion are discussed in Section 4.

## 2. Related work and motivation

### 2.1. Related work

Despite the difficulties described above, many studies in the literature have recently used PET data to automatically segment tumours (15). Techniques range from simple thresholding to advanced machine learning methods (6, 16). “Thresholding,” popular before the deep learning era, relies on clinical experience to define thresholds and can vary greatly between cases, making full automation difficult (2). More refined techniques have been proposed to enhance automation in this process. The majority of these techniques use the distribution of SUV values, like Gaussian Mixtures Models (17) and fuzzy C-means algorithms (18, 19). Others have focused on minimizing a Markov random field (20). However, these models are limited to pixel/voxel-wise segmentation. Another common approach is Active Contour (AC) models, which offer contour-based segmentation and accurate boundary localization (21). Although AC models have the advantage of having the flexibility of topology changes followed by mathematical morphology, these techniques lack a way to work with labelled images in a supervised machine learning framework and often suffer from dealing with noise and occlusions, difficulty in choosing too many parameters, and slow convergence (22).

Recent advancements were influenced by the success of deep Convolutional Neural Networks (CNNs), specifically the U-Net (23) applied to biomedical image segmentation. This is primarily due to their exceptional ability to learn informative hierarchical features directly from data. PET tumour segmentation has also benefited from these developments, for example by using a 3D U-Net to segment brain tumours in PET images (24) and lung tumour segmentation (25–27). However, as illustrated in (28, 29), while skip connections in the U-Net architecture play a role in preserving and integrating detailed information, it does not completely eliminate the inherent challenges in precise boundary recognition due to the information loss in the successive downsampling layers. Despite the headway made in using CNNs for 3D medical image segmentation, their application has been restricted to the generation of voxel-wise segmentation outputs instead of smooth contours. Although CNNs may yield satisfactory segmentation results, low values of the loss function may not always indicate a meaningful segmentation (30). For instance, when there is noise in the input, the resulting background contours may not be accurate. Additionally, classifying voxels near object boundaries can be challenging, particularly in PET images that have low resolution and contrast. Consequently, the boundary delineation may appear fuzzy and imprecise.

### 2.2. Motivation

The task of image segmentation has been closely related to cluster analysis. Clustering methods have become a standard tool for image segmentation due to their ability to group similar image pixels or regions together. Within the clustering framework, the nonparametric or modal formulation is a promising approach for image segmentation. There have been a lot of algorithms developed for the identification of modal regions, which are applied for image segmentation. For example, the mean-shift methods have been applied for a variety of 2D and 3D image segmentation tasks (31–33). Li et al. (34), building on the work by Ray and Lindsay (35) have developed a modal clustering to cluster high dimensional random variables and applied it in natural-scene image segmentation. Compared to most clustering methods, which rely on heuristic similarity measures between objects, nonparametric methods assume that image data is generated from an underlying smoothing process that can be estimated nonparametrically by a probability density. The resulting clusters are defined as the domains of attraction of the density modes.

Nonparametric methods and their ability to identify modal regions have several advantages for PET image segmentation. They are able to handle diverse and complicated image data without making any assumptions about any pre-defined probability density function, which lets them appropriately capture the distribution of the data. In addition, they can identify modal regions of varying shapes and sizes effectively, which aligns with the geometric intuition of clusters, as they are not restricted to a particular shape. This feature makes them useful for segmentation tasks. More specifically, the SUV in PET image often represents the voxel intensity, which can be naturally modelled by probability densities and therefore the tumour regions correspond to the modal regions. Furthermore, the outputs of segmentation are characterized by their spatial continuity, resulting in shapes that may manifest as 2D contours or 3D surfaces. In this work, the 3D spatial structure of the voxels is introduced when building the nonparametric density function – smoothing kernels, to reduce the impact of noise and improve the visual continuity of the SUV distribution. This can aid in identifying and delineating tumour boundaries more accurately, facilitating a more reliable and accurate segmentation process. On the other hand, when two modes are close in proximity relative to the kernel bandwidth, the application of a smoothing kernel might intuitively suggest that the valleys would be further diminished. However, it is important to note that the goal of the smoothing kernel is not solely to separate modes, but rather to improve the overall appearance and compactness of the SUV distribution. The motivation lies in the desire to mitigate the impact of noise and improve the interpretability of the SUV values, leading to more robust and clinically relevant tumour segmentation results.

Another important use of nonparametric methods in PET imaging is the development of IMRT dose painting. In particular, dose painting requires optimising dose distribution based on functional information of the image and can enhance the efficacy of tumour control (14). The notion behind “dose painting” (36) is that functional images can differentiate spatially varying radiation sensitivities of tumours as a basis to “paint” heterogeneous dose prescriptions. One of the popular DP strategies is dose painting by contours (DPBC), which assigns a homogeneous boost dose to the subregions defined by SUV thresholds. As mentioned, the nonparametric methods can model the SUVs as probability densities, which can further aid in capturing the probability mass associated with different metabolic activity levels, enabling a more effective and automated segmentation of the subregions. This probabilistic interpretation, when combined with the level sets, enhances the capability to differentiate and delineate different activity regions in a more robust and informative manner, which in turn can be used to design the IMRT dose painting strategy.

With these motivations, a kernel smoothing-based probability contour (KsPC) approach was proposed in our prior work (37). Instead of a voxel-wise analysis, we assume that the true SUVs come from a smooth underlying spatial process that can be modelled by kernel densities. Further, as the task of segmentation in this case is to distinguish between the tumour and non-tumour region, rather than identifying the modal regions through standard cluster analysis, we have opted to construct an ideal threshold surface to segment out the modal region. The KsPC can provide a manifold over 3D images that naturally produces contour-based results rather than voxel-wise results, thus mimicking experts’ hand segmentation. However, the performance of KsPC depends heavily on the tuning parameters of bandwidth and threshold in the model, and as it is performed per patient, information from other patients cannot be integrated by default. We propose to address these limitations by integrating KsPC in a 3D deep learning framework, which we will call “KsPC-Net.”

## 3. Methodology

In this section, we first illustrate the methodology of our 3D non-parametric density-based segmentation with its 3D probability volume contour development, which formulates the KsPC module in Sections 3.1 and 3.2, respectively. Then we present an integrated CNN framework to embed the KsPC module into a 3D Unet-based architecture with training details in Sections 3.3 and 3.4.

### 3.1. 3D non-parametric density-based segmentation

In this work, we propose to model the 3D voxel-specific SUVs as a discretized version of the underlying unknown smooth process of some “metabolic activity.” The smooth process can then be estimated as the kernel-smoothed manifold of the SUVs over the domain of the entire 3D volumetric images. In particular, let $I=\{p_{1},p_{2},\ldots ,p_{N}\}$ be a 3D volumetric image data, where the ordered set of voxels $p_{i}=((x_{i},y_{i},z_{i}),s_{i}),\;i=1,\ldots ,N$, is described by the vector $(x_{i},y_{i},z_{i})\in V\subset R^{3}$ denoting the coordinates of the voxel’s location, and by the scale quantity $s_{i}$ denoting the SUV or image intensity. We can define a region-of-interest as an open subset $V_{1}$ of V (i.e. V1⊂V), where V is the entire 3D data domain.

We assume that for each voxel $p_{i}$ the SUV represents the frequency that each position vector appears in the corresponding grid. The SUVs can therefore be modelled as kernel density estimate (KDE) (38, 39) of each voxel $p_{i}$ based on the 3D spatial coordinates in a higher dimension as manifold Ψ, which is defined as

(1) $$\hat{\;f}(p;h)=\frac{1}{h_{x}h_{y}h_{z}}{\left(\;\sum_{i=1}^{N}s_{i}\;\right)}^{\;\;-1}\sum_{t=1}^{s_{1}+\cdots +s_{N}}\;K\;\left(\;\frac{x-x_{t}}{h_{x}}\;\right)K\left(\;\frac{y-y_{t}}{h_{y}}\;\right)K\left(\;\frac{z-z_{t}}{h_{z}}\;\right),$$

where K is a kernel function and $h=(h_{x},h_{y},h_{z})$ is the smoothing tuning parameters, called bandwidth which controls the amount of smoothing in each spatial dimension. On the other hand, since $\left(x_{i},y_{i},z_{i}\right)$ is counted $s_{i}$ times at the same position, Equation 1 can be further simplified as

(2) $$\hat{\;f}(p;h)=\frac{1}{h_{x}h_{y}h_{z}}{\left(\;\sum_{i=1}^{N}s_{i}\;\right)}^{-1}\sum_{i=1}^{N}K\left(\;\frac{x-x_{i}}{h_{x}}\;\right)K\left(\;\frac{y-y_{i}}{h_{y}}\;\right)K\left(\;\frac{z-z_{i}}{h_{z}}\;\right)s_{i}.$$

The estimation of the density as in Equation 2 would potentially overcome the limitation of lacking valleys that occur at the border of segments (40). With the spatial coordinates involved in $\hat{\;f}$, the density of a generic voxel depends on voxels that are spatially close to each other. Consequently, at the edge of a segment, where a portion of adjacent voxels exhibits dissimilar SUVs, the resulting density is lower than that of voxels located in the interior of the segment.

Then, in order to achieve an accurate estimation of the density function $\hat{\;f}$, it is necessary to account for two additional factors: the selection of an appropriate kernel function K and the smoothing parameter vector h. With respect to the former, prior research has established that the selection of the kernel function K has a limited influence on the density estimate (41, 42). Hence, for the purposes of this study, we opted for a Gaussian kernel which is denoted as:

$$K(u)=\frac{1}{\sqrt{2\pi }}e^{-\frac{1}{2}u^{2}}.$$

**Figure 2:**
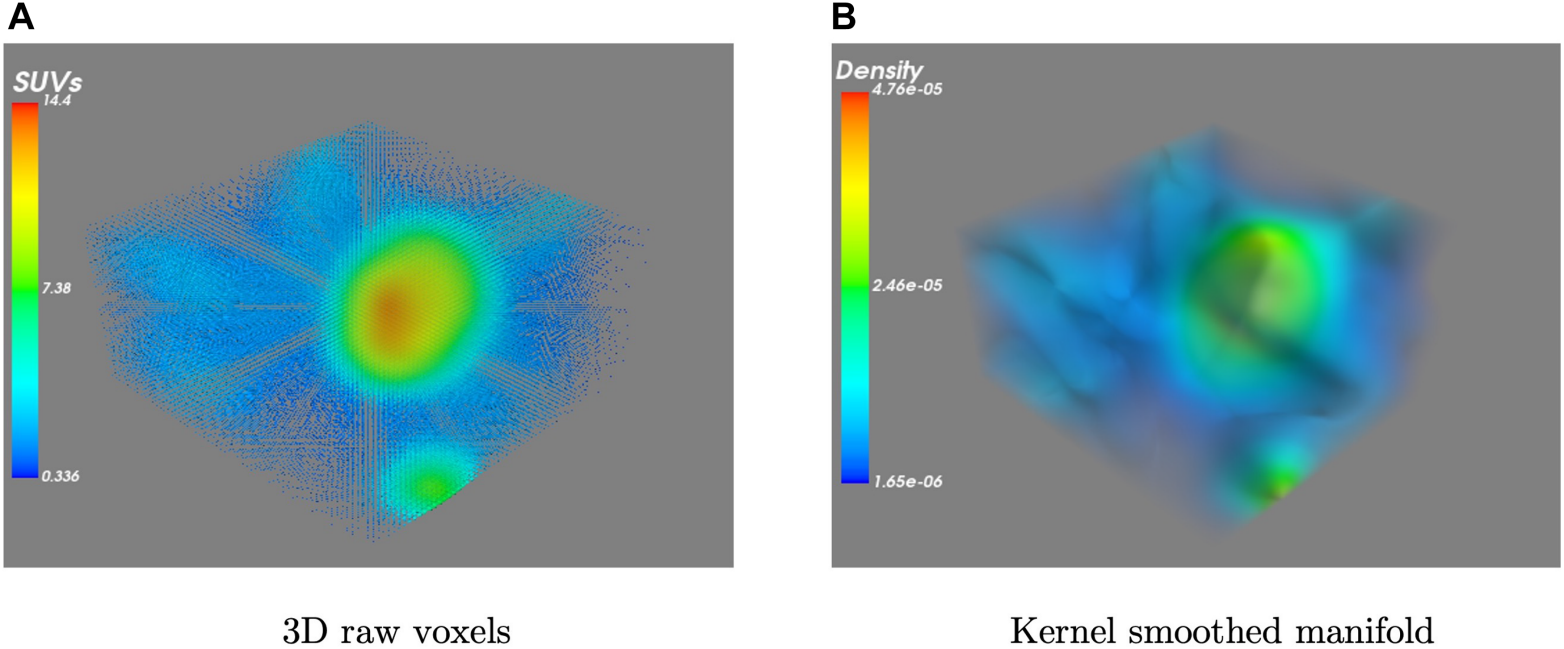
A visualization example of (**A**) a set of raw 3D PET images and (**B**) the resulting kernel smoothed density manifold.

**Figure 3:**
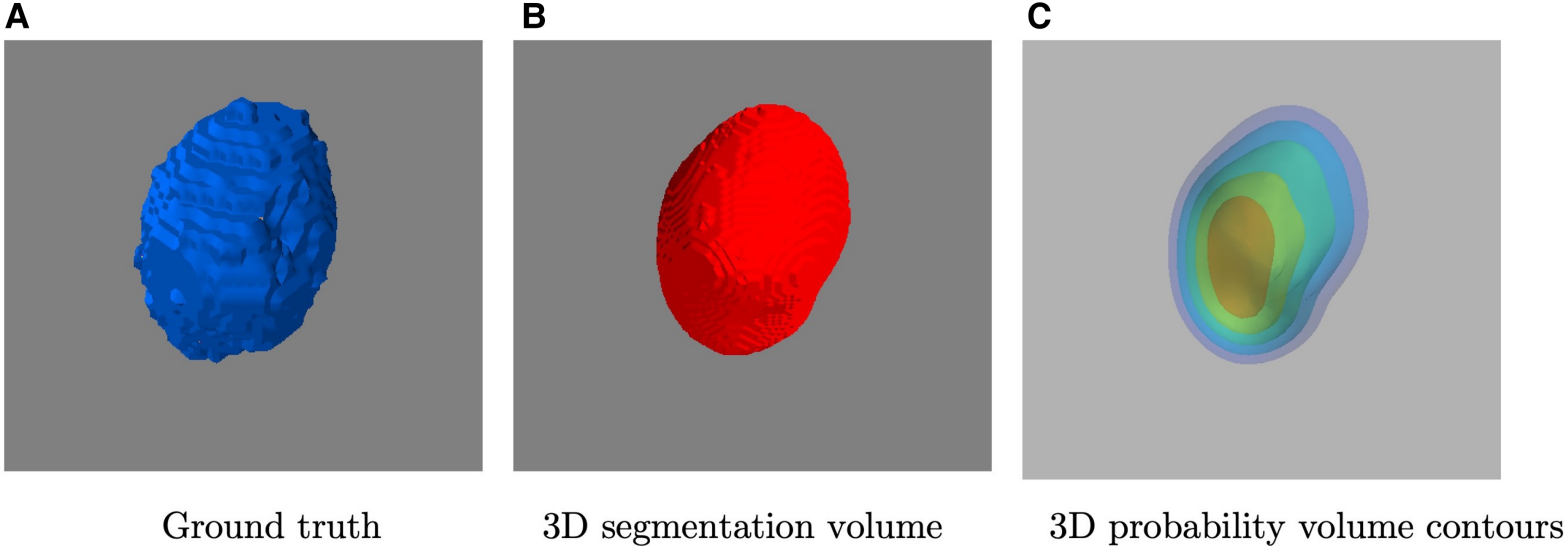
A visualization example of (**A**) ground truth by experts, (**B**) the 3D segmented volume after thresholding and (**C**) its corresponding probability volume contours.

Therefore, we can interpret $\hat{\;f}$ in Equation 2 as the probability mass of voxel p which is estimated by smoothing the SUV values of the local neighbourhood using the Gaussian kernel. Figure 2 presents an example of the original 3D data and its estimated kernel-smoothed manifold. The manifold Ψ is now formed by the estimated density $\hat{\;f}$, and a section of $\hat{\;f}$ at a given threshold λ∈R separates out the region-of-interest as

(3) $$S(\lambda )=\{x\in R^{3}\;:\;\Psi (x)-\lambda =0\},$$

where the $S\in R^{3}$ is the final segmentation surface, which can be viewed as the boundary of the region-of-interest region subset $V_{1}$ (i.e. $S=\partial V_{1}$). An example of the segmented surface can be seen in Figure 3 (B) in comparison with the ground truth provided by experts in (A). Thus, the area inside S denotes the tumour region and the area denotes the background. Note that the tumour region subset $V_{1}$ can be connected or disconnected.

### 3.2. 3D probability contours

After delineating the gross tumour volume, a follow-up application of the kernel smoothed surface is to construct probability volume contours. Mathematically, a 100ω% region of a density f is defined as the level set $S(\lambda_{\omega })=\{x\in R^{3}\;:\;\Psi (x)-\lambda_{\omega }=0\}$ with its corresponding threshold level $\lambda_{\omega }$ such that $P(x\in S(\lambda_{\omega }))=1-\omega$, where x is a random variable and $S(\lambda_{\omega }))$ has a minimal hypervolume (43). In other words, for any ω∈(0,1), the 100ω% surface refers to the region with the smallest area which encompasses 100ω% of the probability mass of the density function (43). In practice, $\lambda_{\omega }$ can be estimated using the following result.

Result 1 The estimated probability threshold level $\lambda_{\omega }$ can be computed as the ωth quantile of $\hat{\;f_{\omega }}$ of $\hat{\;f}(x_{1};h),\ldots ,\hat{\;f}(x_{n};h)$ (Proof in Supplementary Materials).

The primary advantage of utilizing probability volume contours is their ability to assign a clear probabilistic interpretation on the defined volume contours, which are scale invariant (42). This provides a robust definition of probability under the perturbation of the input data. In addition, these contours can be mapped to the IMRT dose painting contours, thus providing an alternative prescription strategy for IMRT. Examples on the application of probability volume contours will be demonstrated and explained in Section 5.

### 3.3. The 3D KsPC-Net architecture

In the KsPC module, the most crucial parameter that determines the performance is the bandwidth (or smoothing parameter) h, as it provides essential information about the locations of high density. In this context, a variety of bandwidth selection techniques have been proposed in the literature on kernel density estimation, including cross-validation and plug-in strategies (42). In our previous work (37) in using 2D KsPC, the bandwidth was determined by cross-validation, being assumed to be the same across spatial dimensions and patients. However, the optimal bandwidth may differ on spatial dimensions and on the patient level, in which scenario the bandwidth selection process can be time-consuming and computationally intensive. Additionally, although the training cost is relatively low in our previous KsPC-only framework, the segmentation process is not fully automated in the sense that prior knowledge of the threshold is learnt specifically for each patient. Furthermore, in regions with low SUV values, the impact of noise and the limited availability of metabolic activity can pose challenges for segmentation algorithms. Therefore, the tumour’s position information plays an important role in accurately locating the relevant regions.

Concerning all these limitations, we propose to integrate the KsPC module with CNN architecture into a unified framework, namely KsPC-Net. Our method is a two-progressive-phase framework for tumour segmentation, which is divided into two stages: coarse segmentation from the CNN backbone and fine segmentation from KsPC. In the coarse segmentation stage, a 3D U-Net architecture-based CNN is employed to provide the bandwidth vector h, tumour position and the threshold needed in the 3D KsPC module. We aimed to leverage the feature extraction capabilities of CNNs to estimate the bandwidth automatically, thereby reducing the need for manual bandwidth selection. In scenarios where the SUV values are very low, the accuracy of the CNN-based segmentation might be compromised while the bounding box information obtained from the coarse segmentation remains valuable. With this bounding box information, we can apply the kernel smoothing method to model the low SUV values within the bounded tumour region. In the fine segmentation stage, the images are segmented in the cropped area and the corresponding probability volume contours are generated through the KsPC procedure.

#### 3.3.1. CNN backbone

**Figure 4:**
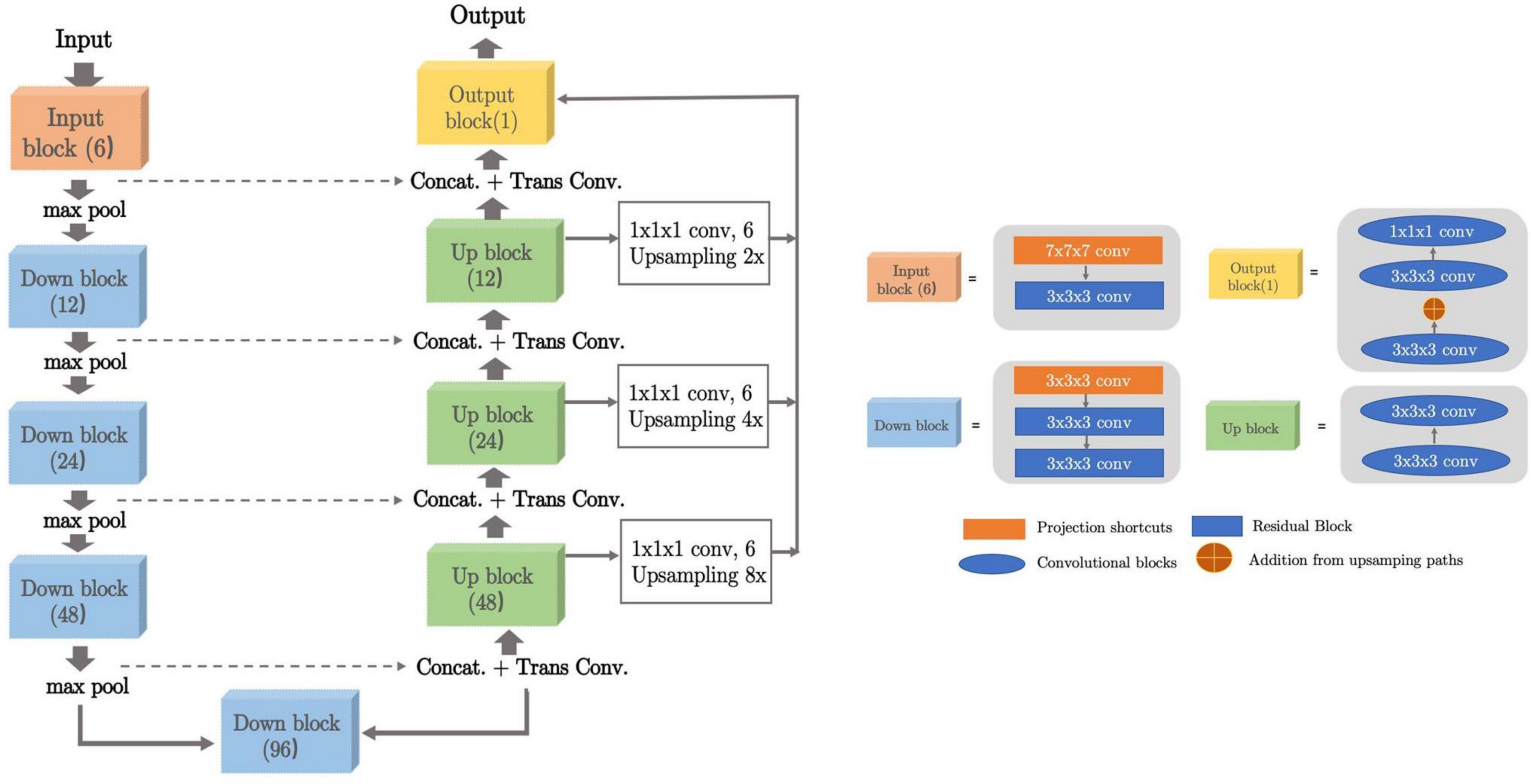
The architecture of 3D LiteSE-Net backbone. The input consists of PET patches of 144×144×144 voxels. Kernel sizes and numbers of output channels are described in each block.

Inspired by the Squeeze-and-Excitation Normalization and nnUNet model (44), we have designed this LiteSE-Net model as our CNN backbone in the coarse segmentation stage. We also denote the original model in (44) as SE-Net, since our proposed LiteSE-Net backbone has a reduced number of channels compared to the original SE-Net. The network structure is shown in Figure 4. The model is built on a classic U-Net architecture (23) with the use of SE Norm layers (45). The input consists of PET patches of 144×144×144 voxels. The encoder consists of residual blocks with identity and project shortcuts. The decoder is formed by convolution blocks. The number of channels in the middle feature map is 6, 12, 24, 48, 96, 48, 24, 12 and 6 respectively. Additional upsampling paths are added to transfer low-resolution features further in the decoder. The details of the definition of the SE Norm layer and projection shortcuts are described in (44).

#### 3.3.2. Coarse-to-fine framework

**Figure 5:**
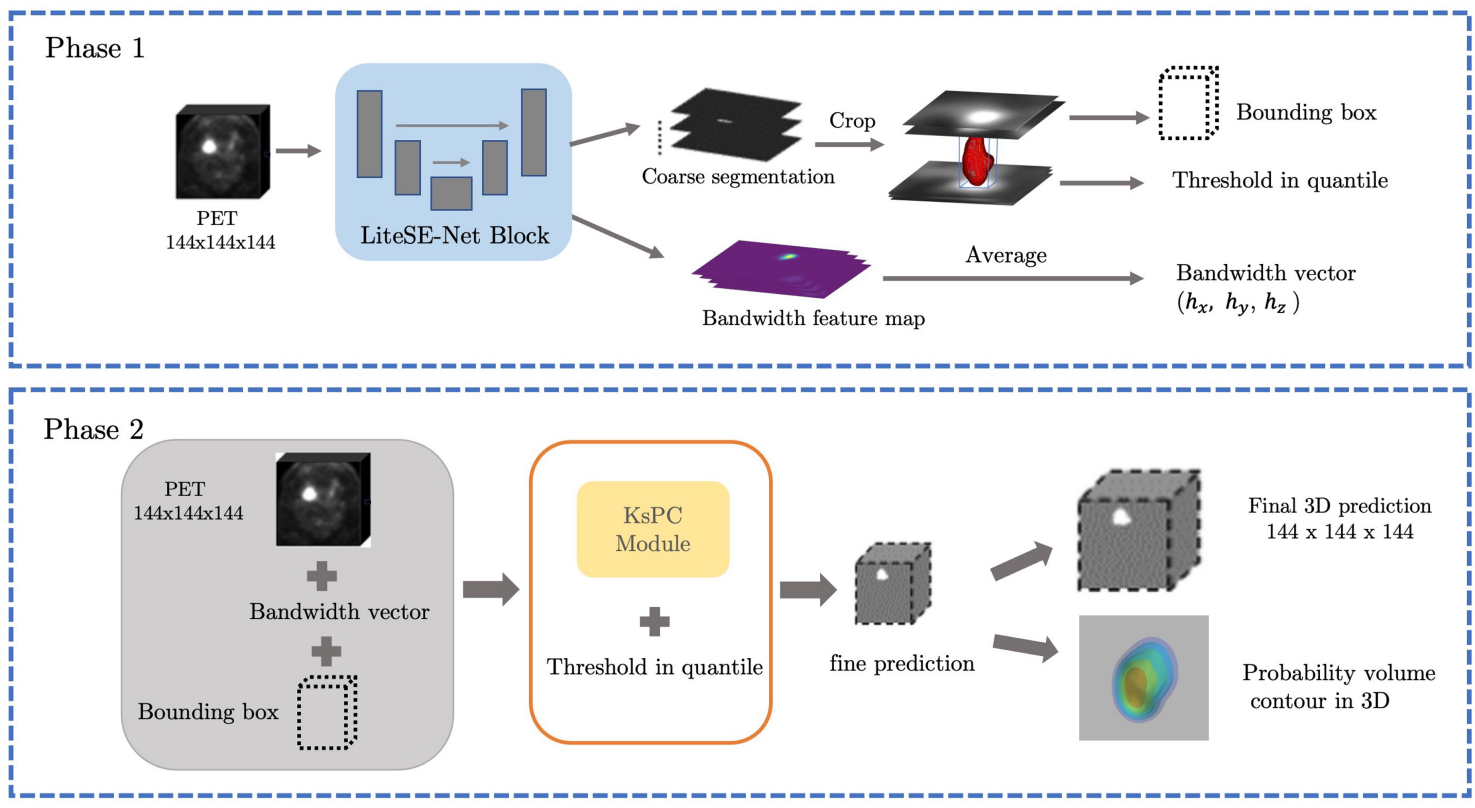
The illustration of our framework. Coarse segmentation phase: a patch of PET images is input into the LiteSE-Net model, and a course segmentation volume is obtained to provide the tumour’s position and a threshold in quantile. In the meanwhile, we take the average of the bandwidth feature volumes to estimate the smoothing vector in each spatial dimension. Fine segmentation phase: the part of the bounding box in PET images is input to the KsPC module with other hyperparameters to get the final segmentation results along with its probability volume contours.

The framework is split into two main phases and illustrated in Figure 5. At the coarse segmentation phase, the initial coarse segmentation prediction and a voxel-level bandwidth feature volume are learned and output by the CNN backbone. The position and size of the tumour along with a threshold in quantile can be obtained from the initial segmentation. More specifically, a cropped bounding box containing the tumour is produced, and the threshold in quantile is computed by identifying the quantile corresponding to the minimum 10% SUV of the tumour region in the initial segmentations. The choice of the minimum 10% SUV is to avoid the influence of any predicted outliers in the coarse segmentation. Additionally, to strike a balance between computational efficiency and performance, we averaged out the bandwidth feature volume in each spatial dimension as the predicted smoothing vector $h=(h_{x},h_{y},h_{z})$. Then, at the fine segmentation phase, we input only the bounding box of PET images into the KsPC module with the predicted smoothing vector and quantile threshold to get the final segmentation volume with its corresponding probability volume contours.

#### 3.3.3. Training scheme overview

**Figure 6:**
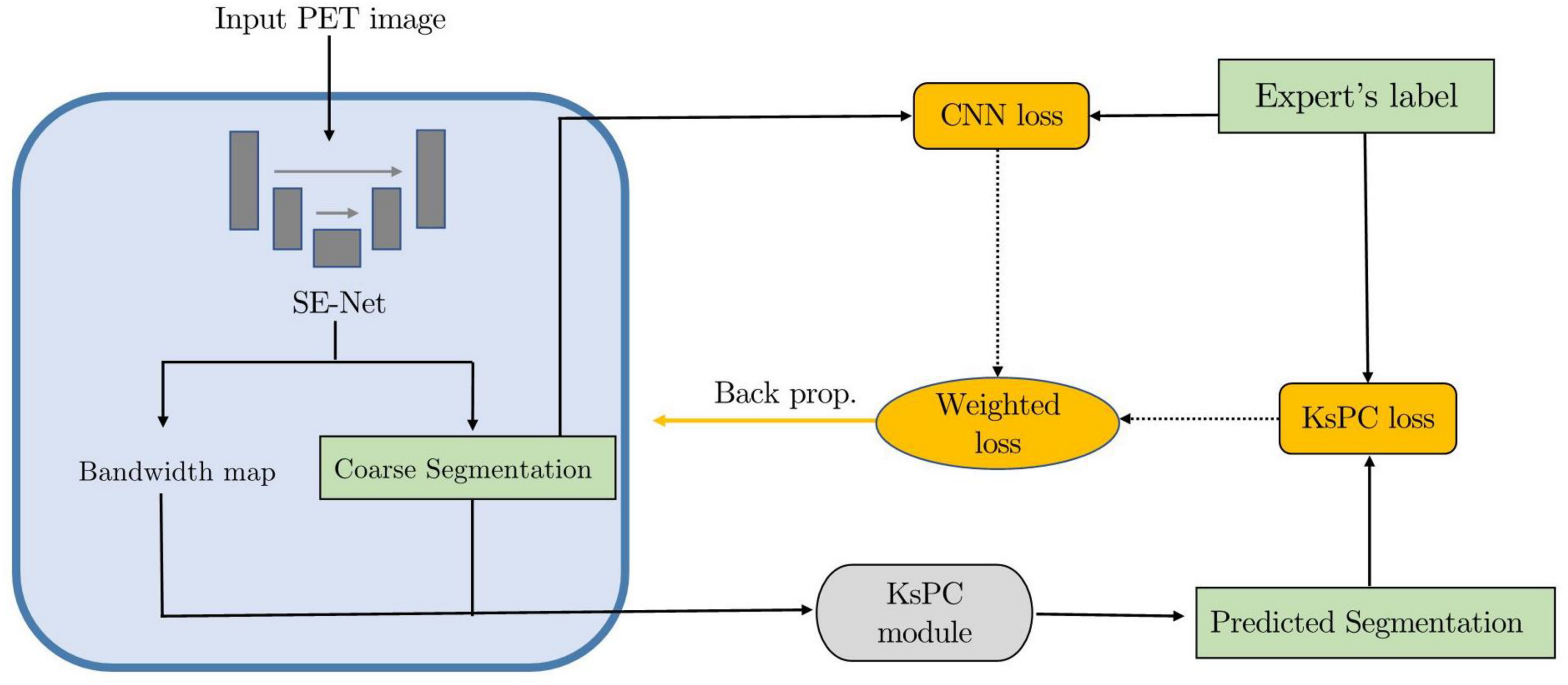
The training scheme of KsPC-Net, an end-to-end trainable framework with KsPC module.

As shown in Figure 6 the proposed KsPC-Net integrates KsPC approach with a CNN backbone in an end-to-end differentiable manner. The resulting output from KsPC is then compared to experts’ labels, referred to KsPC loss. Additionally, the initial coarse segmentation can produce another loss function, called CNN loss, which serves as an auxiliary supervision for the CNN backbone. The final loss can then be constructed as the weighted sum of CNN loss and KsPC loss. By minimizing the final loss, the error can be back-propagated through the entire KsPC architecture to guide the weights updating the CNN backbone.

### 3.4. Model training

#### 3.4.1. Loss function

In order to separate the tumour region completely in the coarse segmentation stage, we hope to increase the recall rate while ensuring the basic segmentation shape to obtain a reasonable threshold. Inspire by Yang et al. (46), We utilize the F-loss function to optimize the model performance during training. The F-loss is defined as followed:

(4) $$\ell_{F}(y,\hat{y})=1-\frac{(1+\beta^{2})\sum_{i=1}^{N}{\hat{y}}_{i}y_{i}}{\sum_{i=1}^{N}{\hat{y}}_{i}+\beta^{2}\sum_{i=1}^{N}y_{i}+\epsilon },$$

where ϵ is set as 1e-8 to avoid the risk of being divided by 0. $\hat{y}$ is the predicted label and y is the ground truth. N is the total number of voxels. The recall rate can be controlled on a reasonable segmentation effect by adjusting the β. When β=1, F-loss is equivalent to Dice loss (47). β is set to be 2 in this paper.

As shown in Figure 6, we construct the weighted loss of two-stage prediction to train the model as follows:

(5) $$\ell_{\mathrm{final}}=\alpha *\ell_{\mathrm{KsPC}}+(1-\alpha )*\ell_{\mathrm{CNN}}$$

where α is a balancing parameter and is set to be 0.7 in this work.

#### 3.4.2. Implementation details

We used Python and a trained network on a Dual Quadro RTX 8000 with 64 GB RAM using the PyTorch package, an open-source deep-learning framework. We applied a batch size of 1 and the Adam algorithm. The cosline annealing schedule was applied to reduce the learning rate from $10^{-3}$ to $10^{-5}$ within every 10 epochs. The proposed model was trained for 100 epochs for best performance and all the comparison state-of-art models were trained for 300 epochs.

## 4. Data and evaluation metrics

### 4.1. Dataset

The dataset is from the HECKTOR challenge in MICCAI 2022 (HEad and neCK TumOR segmentation challenge). The HECKTOR training dataset consists of 224 patients diagnosed with oropharyngeal cancer. The data were acquired from 5 centers with variations in the scanner manufacturers and acquisition protocols (15). In the training dataset, for each patient, FDG-PET input images and corresponding labels in binary description (0 and 1 s) for the primary gross tumour volume are provided and co-registered to a size of 144×144×144 with 1mm×1mm×1mm pixel spacing.

The five-cross-validation is used to generalize the performance of models, where the first step is to split into 5 sets each comprising of 20% of the dataset. The dataset is then systematically tested and evaluated by repeatedly splitting it into training (4 folds comprising 80% of the data) and test sets (1 fold comprising of remaining 20%) multiple times, each time with a different fold as the test set. It is important to note that the validation dataset was independent and not used for model or parameter selection and thus is equivalent to a holdout dataset as it was not seen during the training process. The five-fold cross-validation enhances the robustness of our findings through ensemble models and aggregating results across multiple folds.

### 4.2. Evaluation metrics

For evaluating the performance of the model, we employed the Dice similarity score and Hausdorff distance (HD), which are commonly used as standard ranking criteria in the HECKTOR challenge in MICCAI. Additionally, we reported other metrics such as Precision and Recall for further analysis. The Dice similarity score is a commonly used evaluation metric to assess the performance of segmentation algorithms by evaluating the overlap of the four cardinalities of the confusion matrix, which is defined as

$$\text{Dice similarity score}=\frac{2\mathrm{TP}}{2\mathrm{TP}+\mathrm{FP}+\mathrm{FN}},$$

where TP, FP and FN are the number of true positive, false positive and false negative pixels, respectively. We also compute precision as TP/(TP+FP) and recall TP/(TP+FN) to investigate whether the method was rather providing a large FP or FN rate.

Assessing the performance of a model often hinges on quantifying the differences between contour measures, a task which this paper undertakes by employing the Hausdorff distance (HD) as another key metric. HD is typically characterized as the greatest separation between two distinct structures. Despite its efficacy in capturing the maximal distance between two constructs, the Hausdorff distance is notably affected by minor outliers. To counter this sensitivity, the 95% Hausdorff distance is often employed (15, 48, 49), serving as a more stable estimation of the maximum discrepancy. The 95% Hausdorff distance is generally accepted to represent the 95th percentile of the sorted distance measures as

$${\mathrm{HD}}_{95}(A,B)=P_{95}\left\{\begin{array}{c} \text{sup inf}\;d(a,b),\;\text{sup inf}\;d(a,b)\\ \;a\in A\;b\in B\;a\in A\;b\in B \end{array}\right\},$$

where A is the set for ground truth and B is the predicted volumes. d(a,b) is the Euclidean distance between points a and b, sup and inf are the supremum and infimum respectively. $P_{95}$ is the 95th percentile.

## 5. Results and discussion

### 5.1. Comparisons with other models on Hecktor 2021 dataset

To evaluate the performance of our KsPC-Net, we compared it with results of five-fold cross-validation against three widely-used UNet variant models, namely, the standard 3D UNet (50), the 3D Residual-Net (51) and 3D Dense-Net (52). We also included the original SE-Net model (44), which won first place in the HECKTOR challenge 2020. We evaluated the models using multiple performance metrics, including Dice similarity score, Hausdorff distance, Precision and Recall. The Dice similarity score and Hausdorff distance were the main focus of our assessment, as they provide valuable insights into the accuracy and robustness of methods. The precision and recall were also reported for further analysis. Table 1 shows the quantitative comparison of different models on HECKTOR dataset.

**Table 1:** Mean segmentation results of 3D Unet, Res-Net, Dense-Net, SE-Net and the proposed KsPC-Net, respectively.

Method	Dice score	Hausdf. dist.	Precision	Recall	Learnable parameters
3D-UNet	0.614	18.029	0.664	0,644	6.41M
Res-Net	0.625	7.450	0.685	0.690	8.76M
Dense-Net	0.624	5.767	0.698*	0.634	3.04M
SE-Net	0.646*	6.139	0.675	0.676	9.65M
KsPC-Net(Ours)	0.646*	5.456*	0.637	0.740*	1.36M

Note that the best-performing model for each metric is indicated in bold*.

The results clearly demonstrate that the proposed KsPC-Net is effective in segmenting H&N tumours, achieving a mean Dice score of 0.646. This represents a substantial improvement over standard state-of-art approaches, including 3D-UNet (0.614), Residual-Net (0.625) and Dense-Net (0.624). Our KsPC-Net demonstrates similar levels of Dice scores compared to the SE-Net. However, in terms of Hausdorff Distance, KsPC-Net outperforms all other methods and achieves the best performance, which indicates that KsPC-Net exhibits a stronger capacity for accurately localizing the boundaries of objects. This is consistent with the mechanisms of KsPC, which leverages neighbouring weights to yield outputs with enhanced smoothness. For statistical analysis, our KsPC-Net is significantly better than the standard UNet regarding both Dice scores (p-value=0.023) and Hausdorff distance (p-value<0.00001) while no statistical significance was found compared to the second best-performing method. However, it is important to emphasize that our research objective does not solely focus on attaining the highest level of accuracy in the field. We strive to develop a segmentation framework that not only achieves comparable performance to state-of-the-art models but also enhances stability and interpretability.

Besides the Dice scores and Hausdorff distance, KsPC-Net outperforms all other models with respect to Recall. In comparison to the original SE-Net model, KsPC-Net yields a higher Recall (0.74) with a significant improvement (9.5%), indicating that KsPC-Net generates fewer false negatives (FN). On the other hand, KsPC-Net demonstrates a decrease in precision compared to other methods, potentially leading to over-contouring. This decrease can be attributed, in part, to the utilization of the F-loss function during our experiment. We emphasized recall during the coarse segmentation stage to effectively capture the basic shape of the segmentation. Besides, the presence of false positives (FPs) in areas where PET shows activity but no tumours are present, such as the benign tonsil, can be attributed to physiological activities, inflammatory responses caused by biopsy, and various etiologic causes of infection (53, 54). Additionally, PET’s inherent low spatial resolution can contribute to FPs in the surrounding regions of a tumour (48). Achieving a perfect balance between recall and precision is challenging yet critical to ensure optimal treatment outcomes.

It is also worth mentioning that our goal of the coarse CNN stage is mainly to identify the tumour’s position so the number of channels needed is much lower than in the original SE-Net and all other models. As shown in the last column in the table, our KsPC-Net has a significantly fewer number of learnable parameters than other DL models, which greatly reduces the model’s complexity and training cost. This is due to the much lower number of channels (the maximum is 96) in the feature map in our designed CNN backbone when compares to, for example, the 3D-Unet, Res-Net and Dense-Net are of 256 channels as the maximum in the feature map and SE-Net is of 384 channels.

**Figure 7:**
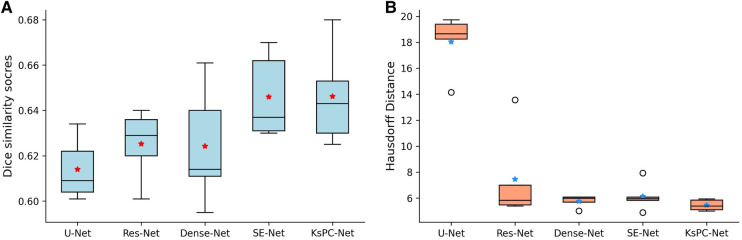
Five cross-validation results on (**A**) Dice scores and (**B**) Hausdorff distance for 3D Unet, Res-Net, Dense-Net, SE-Net and the proposed KsPC-Net, respectively. The average mean is indicated by stars.

In addition, Figure 7 shows the boxplots of the five-fold cross-validation results of each method on Dice score (A) and Hausdorff distance (B). The median value for each model is represented by the horizontal line inside the box. We can see that our proposed KsPC-Net has the highest median Dice score and is slightly higher than the SE-Net though they present the same level of mean. The box represents the interquartile range (IQR), which is the range between the 25th and 75th percentiles of the data. It can be seen that the IQR for our proposed KsPC-Net is relatively small when compared to SE-Net, suggesting less variability. Regarding the comparisons of Hausdorff distance, our KsPC-Net demonstrates a lower median Hausdorff distance. Note that although Dense-Net and SE-Net have slightly less spread-out Hausdorff distance across validation sets, both of them have outliers. This indicates that there might be some extreme cases where the Hausdorff distances are very high in all other methods while the proposed KsPC-Net is able to produce stable and consistent results across different sets.

**Figure 8:**
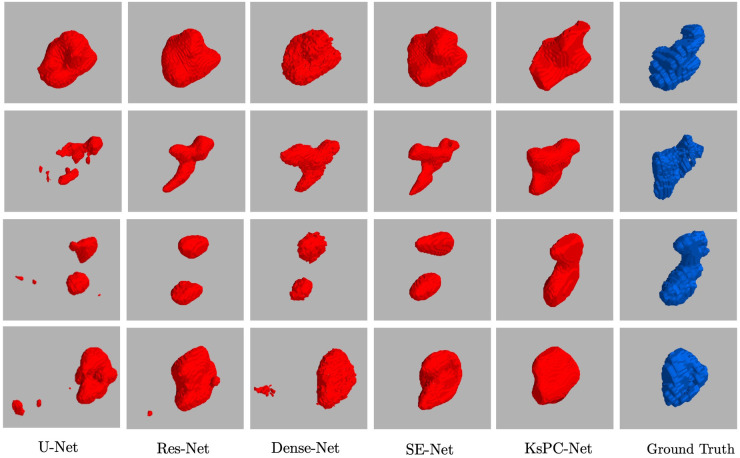
Four representative examples of 3D Segmentation Volumes from 3D Unet, Res-Net, Dense-Net, SE-Net and the proposed KsPC-Net, respectively. The experts’ segmentations are in blue.

In Figure 8, four representative segmented 3D volumes from different models are presented against the expert-segmented ground truth. It can be observed that among all the methods, KsPC-Net consistently outperforms the others across all four test volumes. Specifically, KsPC-Net achieves a more unified segmented volume and a more accurate boundary, particularly in regions with varying shapes. This efficacy stems from the integration of the 3D spatial organization of voxels when formulating the nonparametric density function. Such integration augments the connectivity of modal density regions, thereby enhancing the ability to tackle common challenges in PET images, such as low resolution, low contrast, and noise.

### 5.2. 3D probability volume contours

**Figure 9:**
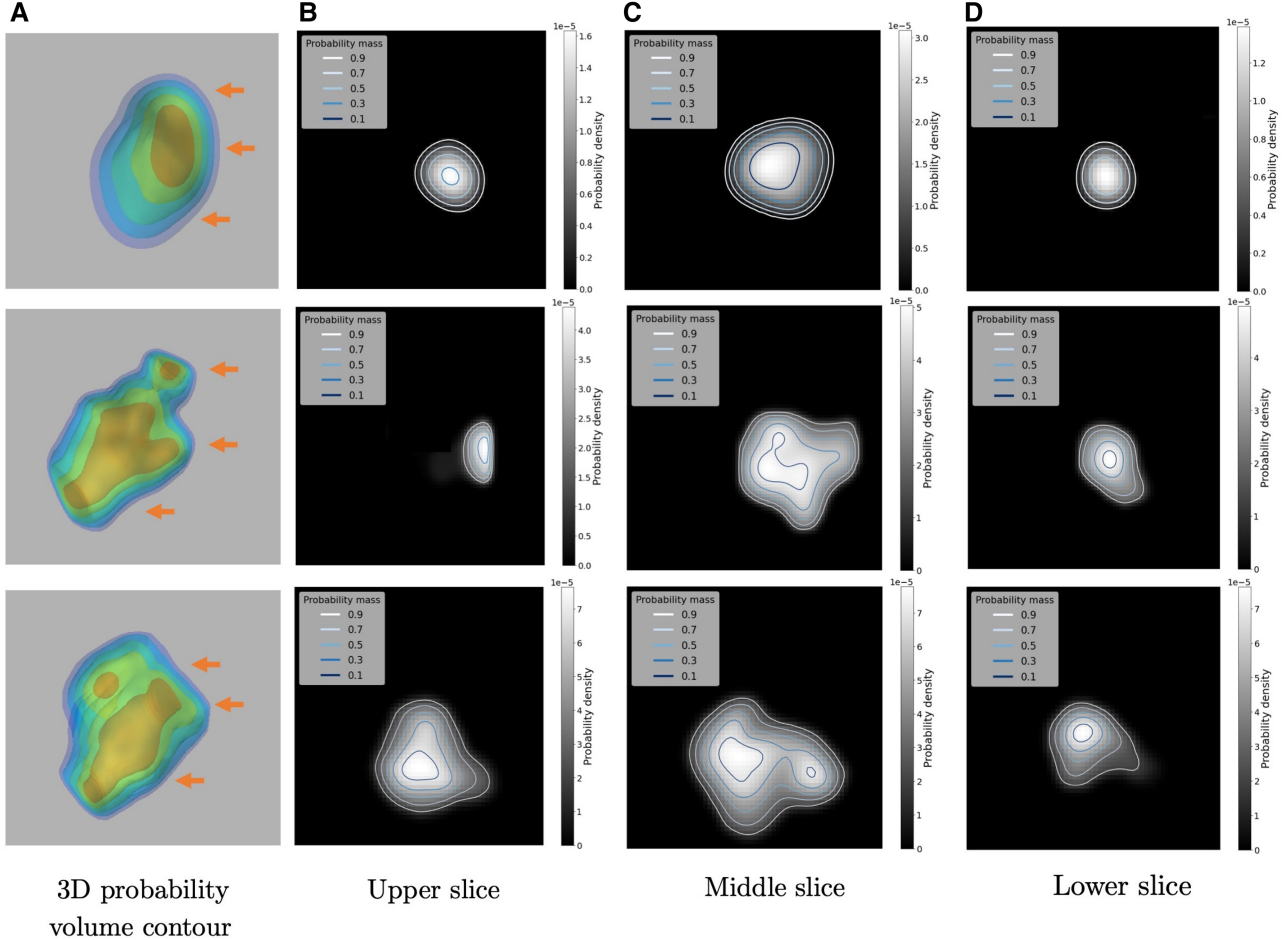
Illustrations of the 3D Probability volume contour and its 2D visualizations on three examples: (**A**) the constructed 3D Probability volume contour (in 10%, 30%, 50%, 70%, 90%). The three orange arrows indicate the upper, middle and lower slicing positions respectively. (**B**–**D**) are the 2D visualizations of the probability contours.

One of the byproducts of using the 3D kernel-smoothed densities to model the SUVs are the associated probability volume contours, which can be readily used to develop a comprehensive inferential framework and can also be used for uncertainty quantification. For example, Figure 9 provides an example of a 3D probability volume contours along with its 2D visualization at different slicing positions, which are denoted by the orange arrows. There are 5 contours in each case which are linear in probability space, in the sense that each contour encloses 10%, 30%, 50%, 70% and 90% probability mass respectively (from inner to outer), thus dividing the density surface into subregions with attached probability mass.

**Figure 10:**
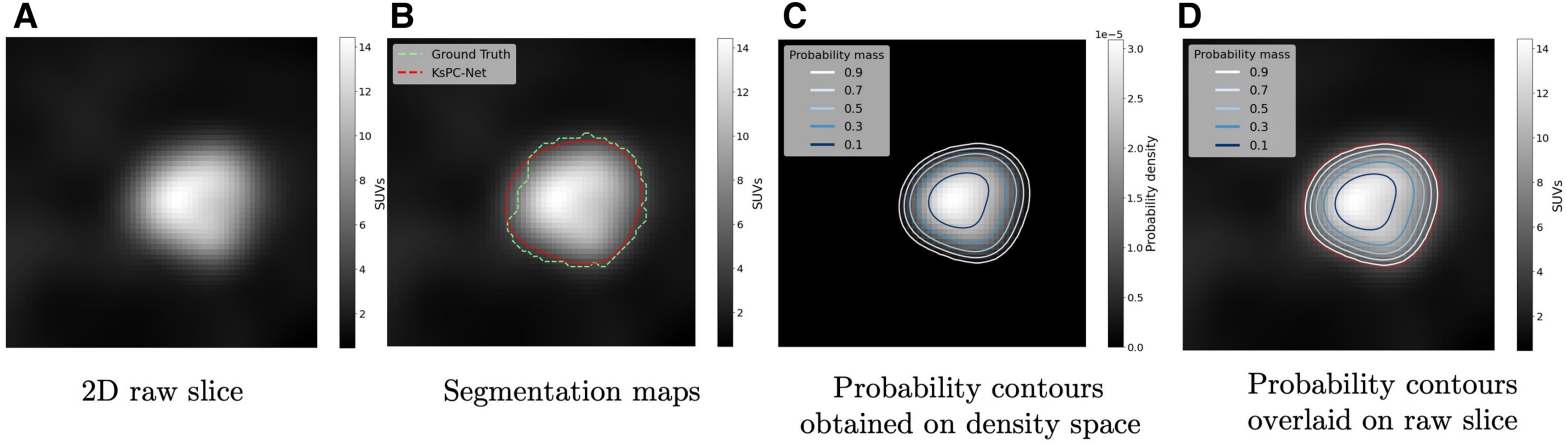
An application of overlaying probability contours onto a raw image. (**A**) The 2D raw slice in SUVs. (**B**) The segmentation contours by KsPC-Net (Red) and expert’s ground truth (Green). (**C**) The corresponding probability contours obtained by KsPC-Net on density space. (**D**) The probability contours (along with segmentation contour) overlaid on the raw slice.

These probability contours can provide a rigorous framework for designing the number and magnitude of SUV thresholds in the optimal dose painting strategies. Since the SUVs are smoothed by the kernel density heights, the inner 10% probability contour corresponds to the subregion with relatively higher SUVs. In other words, there is an inverse mapping between the probability contours and the amount of dose boost assigned to subvolumes. A more detailed example visualized in 2D Region-of-Interest can be seen in Figure 10, where the 2D raw slice is given in Figure 10A. Figure 10B demonstrates the segmentation maps output by KsPC-Net (in red) and the ground truth by experts (in green). Then the obtained probability contours on the density space Figure 10C are superimposed onto the 2D raw slice in SUV scale in Figure 10D.

## 6. Conclusion

In this paper, we present a novel network, KsPC-Net, for the segmentation in 3D PET images with application to Head and Neck tumours, which integrates KsPC (Kernel smoothing-based Probability Contours) into a 3D UNet architecture in an end-to-end differential manner. The KsPC-Net utilizes the benefits of KsPC to deliver both contour-based and grid-based segmentation outcomes, leading to improved precision in segmentation of contours. Promising performance was achieved by our proposed KsPC-Net compared to the state-of-the-art approaches on the MICCAI 2021 challenge dataset (HECKTOR). In terms of computation costs, the proposed model demonstrates a greatly reduced model complexity with a much lower number of channels needed in the DL network. Furthermore, it is crucial to highlight that the main objective of this study is not to develop a new DL method that significantly outperforms existing models in terms of accuracy in measurements. Rather, our primary objective is to provide probability contours as a byproduct alongside the segmentation result while reserving comparable accuracy, which can serve a broader range of applications.

It is worth mentioning that the architecture of our KsPC-Net is not limited to H&N cancer type and can be generalized to a variety of cancer types. Additionally, an important byproduct application of our KsPC-Net is to construct probability contours, which enables probabilistic interpretation of contours. The subregions created by probability contours allow for a strategy planning for the assigned dose boosts, which is a necessity for the treatment planning of radiation therapy for cancers.

There are potential limitations to this work. For example, the segmentation of PET images is often hindered by their low resolution, low contrast, and the presence of noise. Incorporating complementary information is important to obtain a better segmentation. In particular, CT imaging, which is often captured along with PET images can provide additional structural information that can help to define boundaries in PET segmentation more clearly. In light of this, we plan to further develop our model to enable joint segmentation of PET and CT images. By integrating information from both imaging modalities, we aim to improve the accuracy and robustness of our segmentation approach, and ultimately enhance its clinical utility.

## Data Availability

The original contributions presented in the study are included in the article/Supplementary Material, further inquiries can be directed to the corresponding author.
