# Supplementary material for: From coarse to fine: a deep 3D probability volume contours framework for tumour segmentation and dose painting in PET images
Source: Front Radiol. 2023 Sep 5;3:1225215. doi: 10.3389/fradi.2023.1225215 (PMC10512384; doi:10.3389/fradi.2023.1225215)
Supplement: Supplementary file 1 [file Datasheet1.pdf]

Supplementary Material

1 SUPPLEMENTARY PROOF

PROOF OF RESULT1. *Let* $Y = f(\boldsymbol{x})$ *be the density function of the random variable* $\boldsymbol{x}$ *and* f_ω *be the* ω*-th quantile of* Y*, i.e.* $F_Y(f_\omega) = \omega$ *where* F_Y *is the cumulative distribution function of* Y*. Then the* $100\,\omega\%$ *region of the density* f *is given by*

$$
\begin{aligned}
\mathcal{P}(\boldsymbol{x} \in \mathcal{L}(f_\omega) = \int_{\mathcal{R}^d} f(\boldsymbol{x})\,\boldsymbol{1}\,\mathcal{L}(f_\omega)d\boldsymbol{x} = E\{\boldsymbol{1}\{f(\boldsymbol{x}) \geq f_\omega\}\} \\
= \mathcal{P}(f(\boldsymbol{x}) \geq \omega) = \mathcal{P}(Y \geq \omega) \\
= 1 - F_Y(f_\omega) = 1 - \omega \ .
\end{aligned}
$$

2 SUPPLEMENTARY TABLES

The five-fold cross validation results for each model are presented here.

Table S1. Five-fold cross validation for 3D U-Net

Unet	Dice Score	Hausdf. Dist	Precision	Recall
Fold1	0.601	19.390	0.634	0.635
Fold2	0.609	18.643	0.668	0.610
Fold3	0.634	14.142	0.638	0.693
Fold4	0.604	18.240	0.678	0.616
Fold5	0.622	19.730	0.706	0.666

Table S2. Five-fold cross validation for 3D Res-Net

Res-Net	Dice Score	Hausdf. Dist	Precision	Recall
Fold1	0.636	5.477	0.701	0.643
Fold2	0.601	7.000	0.691	0.597
Fold3	0.640	5.830	0.694	0.678
Fold4	0.629	5.385	0.709	0.641
Fold5	0.620	13.558	0.655	0.670

Table S3. Five-fold cross validation for 3D Dense-Net

Dense-Net	Dice Score	Hausdf. Dist	Precision	Recall
Fold1	0.614	5.986	0.678	0.618
Fold2	0.595	6.077	0.679	0.606
Fold3	0.611	6.082	0.705	0.662
Fold4	0.640	5.682	0.690	0.662
Fold5	0.661	5.012	0.740	0.638

Table S4. Five-fold cross validation for 3D SE-Net

SE-Net	Dice Score	Hausdf. Dist	Precision	Recall
Fold1	0.631	5.830	0.653	0.686
Fold2	0.630	5.958	0.694	0.620
Fold3	0.662	6.082	0.677	0.718
Fold4	0.637	4.898	0.642	0.680
Fold5	0.670	7.928	0.711	0.680

Table S5. Five-fold cross validation for 3D KsPC-Net

KsPC-Net	Dice Score	Hausdf. Dist	Precision	Recall
Fold1	0.630	5.385	0.648	0.696
Fold2	0.625	5.854	0.651	0.684
Fold3	0.643	5.944	0.603	0.780
Fold4	0.653	5.099	0.635	0.763
Fold5	0.680	5.000	0.650	0.777
